# Methods for Developing Evidence Reviews in Short Periods of Time: A Scoping Review

**DOI:** 10.1371/journal.pone.0165903

**Published:** 2016-12-08

**Authors:** Ahmed M. Abou-Setta, Maya Jeyaraman, Abdelhamid Attia, Hesham G. Al-Inany, Mauricio Ferri, Mohammed T. Ansari, Chantelle M. Garritty, Kenneth Bond, Susan L. Norris

**Affiliations:** 1 Knowledge Synthesis Platform, George & Fay Yee Center for Healthcare Innovation, Faculty of Health Sciences, University of Manitoba, Winnipeg, Manitoba, Canada; 2 Department of Community Health Sciences, College of Medicine, Faculty of Health Sciences, University of Manitoba, Winnipeg, Manitoba, Canada; 3 Department of Obstetrics and Gynecology, Faculty of Medicine, Cairo University, El-Manial, Cairo, Egypt; 4 World Health Organization, Geneva, Republic and Canton of Geneva, Switzerland; 5 Clinical Epidemiology Program, Ottawa Hospital Research Institute, Ottawa, Ontario, Canada; 6 Canadian Agency for Drugs and Technologies in Health, Ottawa, Ontario, Canada; University of Cambridge, UNITED KINGDOM

## Abstract

**Introduction:**

Rapid reviews (RR), using abbreviated systematic review (SR) methods, are becoming more popular among decision-makers. This World Health Organization commissioned study sought to summarize RR methods, identify differences, and highlight potential biases between RR and SR.

**Methods:**

Review of RR methods (Key Question 1 [KQ1]), meta-epidemiologic studies comparing reliability/ validity of RR and SR methods (KQ2), and their potential associated biases (KQ3). We searched Medline, EMBASE, Cochrane Library, grey literature, and checked reference lists, used personal contacts, and crowdsourcing (e.g. email listservs). Selection and data extraction was conducted by one reviewer (KQ1) or two reviewers independently (KQ2-3).

**Results:**

Across all KQs, we identified 42,743 citations through the literature searches. KQ1: RR methods from 29 organizations were reviewed. There was no consensus on which aspects of the SR process to abbreviate. KQ2: Studies comparing the conclusions of RR and SR (n = 9) found them to be generally similar. Where major differences were identified, it was attributed to the inclusion of evidence from different sources (e.g. searching different databases or including different study designs). KQ3: Potential biases introduced into the review process were well-identified although not necessarily supported by empirical evidence, and focused mainly on selective outcome reporting and publication biases.

**Conclusion:**

RR approaches are context and organization specific. Existing comparative evidence has found similar conclusions derived from RR and SR, but there is a lack of evidence comparing the potential of bias in both evidence synthesis approaches. Further research and decision aids are needed to help decision makers and reviewers balance the benefits of providing timely evidence with the potential for biased findings.

## Introduction

Healthcare decision- and policy-makers around the world depend greatly on evidence to inform and guide decisions made at the bedside to those made at the level of Health Ministries. Systematic reviews “attempt to collate all empirical evidence that fits pre-specified eligibility criteria in order to answer a specific research question” [1]. As a result of this comprehensiveness and rigor, they are generally acknowledged worldwide as providing the most trustworthy scientific evidence to support both clinical and policy decision-making. Advantages of systematic reviews include increasing statistical power and decreasing the likelihood of type II errors, minimising the influence of bias from primary literature, following an a priori protocol, and transparently reporting review methods.

While the benefits of systematically summarizing and evaluating scientific evidence are well-known and accepted, not all organizations have the resources to invest in conducting systematic reviews or the luxury of waiting for the results before making a decision. Additionally, there is no one systematic review methodology that is accepted by all as the most rigorous. To complicate matters further, not all ‘systematic reviews’ are high quality nor do they follow a certain methodology [2]. From an academic perspective, short-cutting systematic review methods, or poor reporting of the methods used, is generally unacceptable [3]. Even so, feasibility, resources and timelines are important considerations.

The World Health Organization (WHO), among other organizations, must often formulate clinical or public health policy quickly when certain events occur that lead to urgent, newly-identified needs. Such an event could be, for example, a natural disaster, warfare, wide-spread biologic or chemical exposures, or an unforeseen disease epidemic (e.g. Ebola outbreak [4]). The WHO Guideline Review Committee (GRC) considers Rapid Advice Guidelines as those produced “in response to a public health emergency” (such as pandemic influenza [5]) for which “WHO must provide global leadership and timely guidance in the form of an evidence-informed guideline produced within one to three months” [6].

When guidance must be issued within these time constraints, comprehensive systematic review methods may not be feasible. As a consequence, guidance may have to be based only on the results of a very limited or abbreviated review process. This may include recommendations based on evidence from available systematic reviews and guidelines, recent trial reports, and/or expert opinion.

The need for more rapidly produced evidence summaries is ever-increasing, which is reflected by the increasing number of rapid reviews being produced. Even so, rapid reviews, unlike systematic reviews, lack a single agreed-upon definition and standard, reliable, valid, and high-quality methods. Additionally, rapid reviews appear to be less frequently published in academic journals compared with systematic reviews answering similar questions. This may be due to a belief among academics that the evidence provided by rapid reviews is inferior to that provided by systematic reviews and not of the same academic merit.

There has been increasing interest in producing evidence to support decision-making in a short period of time. Researchers have begun to document, index and classify emerging trends to rapid evidence synthesis [7–13]. This interest in rapid evidence synthesis is not only limited to academics, but also to decision-makers as well [14,15].

Therefore, the objective of this study was to systematically search for, identify, and summarize the (a) methods currently used by organizations around the world for producing rapid reviews; (b) evidence on the reliability, validity, and quality of rapid reviews compared with systematic reviews; and (c) sources of bias associated with rapid reviews. Even though the definition of “rapid” in this context varies from organization to organization, we were interested in evidence summaries that were produced within three months or less by limiting the topic scope or review methods used or by deviating from accepted standards for systematic review production [1,16,17]. The ultimate goal was to inform the WHO GRC about which processes and procedures might be reasonable to modify when developing evidence summaries to support Rapid Advice Guidelines.

## Methods

### Search strategy for identification of publications

As this systematic review spanned several separate but closely related key questions (KQ), we prepared separate search strategies to answer each question. We used an a priori protocol “S1 Text” and the search strategies were developed by a team of experienced researchers and information specialists, and were peer-reviewed [18].

KQ1: What methods are used by organizations producing rapid reviews?

In order to gather information on methods used by organizations to conduct rapid reviews, we undertook a multi-stage searching process including a scoping review of published rapid reviews and a search of organizations commissioning or conducting rapid reviews as identified by web searches, social media, and personal contacts, including snowballing. We chose a scoping review methodology to initially map key published rapid reviews and identify a preliminary list of organizations conducting such reviews. We began by conducting a scoping search to identify rapid reviews in MEDLINE^®^ (Ovid), EMBASE^®^ (Ovid), and Cochrane Library (Wiley) “S1 Table”. The searches were limited to English language articles published since the year 1980. The date restriction was used for feasibility and it was not anticipated that searching prior to this date or in additional languages would bias the results because (1) most citations and core journals indexed in MEDLINE^®^, EMBASE^®^ and the Cochrane Library are published in the English language; (2) most organizations known to regularly conduct systematic reviews publish some version of their reports in the English language; and (3) accepted standards for the conduct of systematic reviews have changed markedly over the years, so evidence published prior to this date would likely be outdated. In addition, methods for “systematic reviews” were not developed by organizations like Cochrane until the early 1990’s; and even they are in a constant phase of evolution.

KQ2: What is the reliability, validity, and quality of rapid reviews?

To identify studies that assessed the reliability, validity or quality of rapid versus systematic reviews, we used a similar search strategy as presented for KQ1 but limited the search to comparative studies “S2 Table”.

KQ3: What are the potential biases associated with rapid reviews?

To identify studies that investigated and/or reported empirical evidence of bias in the findings of rapid reviews, we used a series of searches in MEDLINE^®^ (Ovid), EMBASE^®^ (Ovid), and Cochrane Library (Wiley) “S3 Table”. As we anticipated limited research to have been conducted on systematic errors (bias) in rapid reviews, we began by identifying evidence of bias that can be introduced into systematic reviews. Further, since we knew that secondary research had already been published on this topic, we began with a search for systematic reviews since 1980 and supplemented that with searches for recent primary literature from 2010 onwards.

For all key questions, we also searched for unpublished and non-indexed documents through personal contact and consultation with experts, soliciting citations and documents on social media (e.g. LinkedIn Evidence-Based Medicine groups), via an Email listserv (evidence-based-health@jiscmail.ac.uk), and using a snowball sampling technique. In addition, we searched the Grey Matters [19] list and the websites of organizations that were known to conduct rapid reviews. The reference lists of relevant articles were also searched for relevant citations.

Literature screening employed standardized piloted screening forms. For KQ1 we adopted the scoping review methodology [20–22] and employed a single reviewer literature screening approach [AMAS]. For KQ2-3, we used a two-stage process for study screening and selection. Two reviewers [AMAS, MJ, MF, HAI, MF] independently screened the titles and abstracts of retrieved records to determine eligibility. The full text of citations classified as “include” or “unclear” were reviewed in detail to confirm eligibility. Discrepancies between the two reviewers were resolved through discussion and consensus.

### Study selection

KQ1: What methods are used by organizations producing rapid reviews?

We included published rapid reviews (author-defined), conducted to inform decision-making within an organization, and using current methods. We considered methods to be current if the review was published within the last five years or methods are still being used (confirmed by online documentation or via personal contact). In the event we found older articles of interest, we attempted to contact the corresponding author or organizational representative to determine if the methods described were still being used.

Further, using a compiled list of organizations known to have produced rapid reviews, we searched online for documentation of their rapid review methods. If none were identified, we attempted to contact the organization for more details. In addition, we collaborated with researchers already collecting similar, but not identical, data on rapid review methods to support the creation of a Cochrane Methods Group dedicated to rapid reviews, the Rapid Reviews Methods Group (http://methods.cochrane.org/news/rapid-reviews-methods-group) [23].

KQ2: What is the reliability, validity, and quality of rapid reviews?

The inclusion criteria were studies comparing rapid with systematic reviews, and reporting on the comparative reliability, validity and/or quality of the evidence summaries.

KQ3: What are the potential biases associated with rapid reviews?

The inclusion criteria were systematic reviews and primary studies investigating potential biases that may be introduced as a result of deviating from the accepted systematic review methods.

### Data abstraction and management

A single reviewer extracted relevant data using standardized and piloted data extraction forms; while a second reviewer verified the extracted data for completeness and accuracy. Discrepancies between the reviewers were resolved by discussion and consensus. A single reviewer documented information received via personal contact.

### Data analysis

KQ1: What methods are used by organizations producing rapid reviews?

Rapid review methods were classified and categorized to generate a map of the methods used worldwide. We calculated frequencies and density distributions in order to determine prevalence and trends in the data. Results are reported in aggregate and anonymously as some of the information was received through personal contact.

KQ2: What is the reliability, validity, and quality of rapid reviews?

We summarized the objectives, methods used, and results of studies comparing rapid with full reviews. No meta-analytic techniques were used.

KQ3: What are the potential biases associated with rapid reviews?

We classified and categorized identified potential biases narratively and in tabular form. No meta-analytic techniques were used.

Reference management was conducted using EndNote™ (version X5, Thomson Reuters, Carlsbad, CA, USA). Screening and data management was performed using Microsoft Excel™ 2010 (Excel version 14, Microsoft Corp., Redmond, WA, USA).

## Results

KQ1: What methods are used by organizations producing rapid reviews?

Following screening of 17,713 citations gathered from the literature search “S2 Text”, we identified 65 organizations producing rapid reviews “S1 Fig”. We were unable to identify methods used currently by all these organization, but did manage to identify methods used by 29 organizations. These organizations produced 33 different rapid review report types; three organizations produced multiple rapid review types, each with its own unique methods and timelines.

Organizations conducting rapid reviews were distributed globally with the majority in North America (n = 16) and Europe (n = 9) and were mainly governmental (n = 12) or societies/ independent (n = 11) organizations, with a minority (n = 6) being associated with academic institutions “Table 1”. Rapid reviews were reported to be conducted on average in 3.2 months (range: 0.5 to 12 months; median: 3 months, IQR: 1.75 to 4 months).

**Table 1:** Geographic distribution of organizations conducting rapid evidence synthesis with methods available for review.

Country	Number of organizations
Australia	3
Austria	1
Canada	12
Finland	1
Germany	1
Italy	1
Sweden	1
Taiwan	1
UK	4
USA	4

Diverse context- and organization-specific rapid review approaches were identified. Topic selection criteria, documenting methods in an a priori protocol, general literature search strategies, and policies for reviewers on how to conduct study selection, data extraction, evidence synthesis and preparing an evidence report were generally well-developed within the individual rapid review methods, but there were marked differences among the organizations.

Despite their uniqueness, we observed some general trends among the reported methods “Table 2”. For example, informing/supporting decision-making was one of the top priorities for undertaking rapid reviews. Similarly, publication of the final report in peer-reviewed journals was rare. At the same time, most organizations reported publishing their final reports online in some form (e.g. organization website, social media, etc.).

**Table 2:** Summary of methods used by organizations in conducting rapid evidence synthesis.

**Background**	
Rapid review definition:	Variable; usually carries two important concepts: “need to be timely/ efficient” and “use of limited systematic review methodology”. Often these two observations are coupled to express a trade-off between being “rapid” and being “comprehensive”.
Average time to complete (months):	Mean (SD): 3.2 months (2.3 months); Median (IQR): 3.0 months (1.8 to 4.0 months); Range: 0.5 to 12 months.
**Topic selection**	
Official intake/ review process:	Most organizations (81%) have an official intake/ review process for prospective requests for rapid reviews. The reviewers may provide possible tweaks on the original question, but cannot usually propose an alternate topic. Some groups ranked requests for importance to assist in the intake process.
Type(s) of research question(s) rapidly reviewed:	Variable; ranged widely, based on organizational needs, but includes clinical efficacy, effectiveness, safety, and cost-effectiveness.
Main focus of the final review report:	Inform/ support patients, clinicians and decision-makers in some capacity (100% of organizations).
**Protocol**	
A priori protocol:	Protocols commonly prepared (84%), but rarely registered (n = 4; 16%).
**Literature search**	
Databases regularly searched:	Most searched at least 3 bibliographic databases, but numbers varied markedly. Most identified “PubMed/ Medline”, “Cochrane Library” and “EMBASE” as the most frequently searched.
Identifying previous systematic reviews and primary studies:	Previous systematic reviews were frequently searched (100%) along with recent primary studies (78%). Some groups only search for systematic reviews, while others search for primary studies if no systematic reviews are identified.
Search limits:	Most common limits were for publication year (63%), language (72%) and study design.
Grey literature searching:	Grey literature is variably searched (56%).
Peer-reviewing search strategies:	If conducted, usually performed internally within the organization (38%).
**Study selection**	
Number of reviewers selecting studies:	Variable; different groups used only a single reviewer (41%), one reviewer checks the decisions made by another reviewer (9%), two independent reviewers (38%), or one to two reviewers (project-specific).
**Data extraction**	
Number of data extractors:	Variable; different groups used only a single reviewer (41%), one reviewer checks the decisions made by another reviewer (25%), two independent reviewers (22%), or one to two reviewers (project-specific).
Data extracted from included studies:	Study characteristics, population demographics, intervention/ comparator characteristics, and outcome measures (numerical and categorical data) usually extracted. Outcomes measures often limited by scope of review, or set a priori.
**Evidence synthesis**	
Summary evidence presentation:	Narrative summary (most frequent; 91%), but may include vote-counting and/ or meta-analysis (less frequently; 6%).
Critical appraisal of the internal validity of the included studies:	Variable: different groups used only a single reviewer, one reviewer checks the decisions made by another reviewer, two independent reviewers, or one to two reviewers (project-specific). Some groups do not conduct critical appraisal. No consensus on which tool(s) to use.
Study author/ industry contact for missing data, unpublished trials, or feedback:	Infrequently performed by any organization (25%).
**Evidence report**	
Items presented in final review report:	Variable: usually contains “recommendations”, “implications for policy”, and “an explanation of the strengths/ weakness of the review methods used (e.g. disclaimer)”.
Peer-review:	Some sort of peer-review usually conducted (94%), but often limited to internal review and feedback from requestor; variable external peer-review.
Final report dissemination:	Final report frequently disseminated to individuals other than the requestor (88%).
**Dissemination**	
KT/ dissemination tools:	Variable: organization-specific, but conclusions of most rapid reviews are posted online in some form (e.g. organizational website, social media) (72%).

Most organizations searched PubMed/MEDLINE^®^, the Cochrane Library and EMBASE (less frequently); other bibliographic databases were searched less frequently. Searches were often limited by publication year, language, and study design. Organizations varied in their searching of grey literature, and the decision to do so was often topic specific.

Readers of systematic reviews generally expect duplication of tasks by at least two independent reviewers and to have conflicts resolved by discussion and consensus or by adjudication by a third reviewer [1]. In contrast, organizations conducting rapid reviews varied in their use of duplication during study selection and data abstraction: some used two independent reviewers, some had a second reviewer verify the decisions made by the first reviewer, some used a flexible model whereby any degree of duplication was based on resource availability and the topic being reviewed, and some did not use duplication at all, choosing instead to have only a single reviewer. Furthermore, authors of research being reviewed were rarely contacted for clarification or missing information. Most methods included some form of study validity/risk of bias assessment of the included research, but the tools being used for these assessments varied among organizations. While quantitative evidence was usually summarized for the readers using a narrative approach, some organizations summarized the rapid review results using vote counting or meta-analytic techniques. Finally, reporting guidelines and dissemination tools were organization specific and most organizations conducted internal peer review/discussion among organizational members about the results of the rapid evidence summary rather than seeking external peer review.

KQ2: What is the reliability, validity, and quality of rapid reviews?

Following screening of 6,528 citations gathered from the literature search, 10 records [24–33] (nine primary studies[24–32] and one companion publication [33]) were identified comparing rapid versus systematic reviews and health technology assessments “S2 Fig”.

Summaries of the included studies revealed two important observations “Table 3”. First, the majority of rapid reviews in the comparisons were conducted by governmental organizations to support time-sensitive decision-making. In contrast, the systematic reviews were conducted by academics who published their results in peer-reviewed journals. Secondly, there was general agreement that the methods used by rapid and systematic reviews/health technology assessments differed in several aspects, with rapid reviews often reporting the methods used in much less detail.

**Table 3:** Summary of studies comparing systematic reviews and rapid.

Study	Methods	Results
**Cairns 2006[24]**	Comparison of 21 rapid reviews conducted by SMC vs. systematic review by NICE.	Methods used by SMC and NICE are markedly different. Even so, main conclusions (accept/ reject new medicines or technology) were similar in most reviewed cases (20/ 21).
**Cameron 2007[25,33]**	Comparison of seven rapid vs. four systematic reviews conducted independently by different groups.	Methodology of rapidly reviewing the evidence differed between rapid reviews reviewed, and often under-reported. Scope is narrower for rapid reviews, but main conclusions often don’t differ.
**CADTH 2012[26]**	Comparison of one rapid review conducted by Canadian Agency for Drugs and Technologies in Health vs. systematic review conducted by Technology Assessment Unit (McGill University).	Methods used by the rapid review and the full review differed in several aspects. Even so, the conclusions of both reviews were similar.
**Kaltenthaler 2008[27]**	Comparison of two rapid NICE STAs vs. one full systematic TAR for the same healthcare technologies.	Methods used by STAs require more dependence on data submitted by industry with limited capacity for additional searching for data. Conclusions were similar, but sources of evidence were variably absent in the STA assessments.
**Lopez 2003[28]**	Comparison of one journal-published rapid review vs. one journal-published systematic review.	Methods used by rapid review were abbreviated as compared with the systematic review. This led to identifying only a fraction of the available trials found by the full review (3/ 44). Even so, main conclusions were not different, but differences were evident in identified associations and confounding factors.
**Peinemann 2008[29]**	Comparison of rapid review conducted by IQWiG and systematic reviews.	Methods were inconsistent across systematic reviews and showed some similarities/ differences with the IQWiG rapid review. Trial identification was not only different due to differences in search criteria and sources of evidence, but also study included study designs.
**Saz Parkinson 2010[30]**	Comparison of rapid vs. exhaustive health technology assessments.	Both types of HTA reports offer similar information for decision-makers, especially when presenting cost-effectiveness analysis, ethical and legal impacts, or exploring critical uncertainties and proposals for clarifying them.
**Van de Velde 2011[31]**	Comparison of one journal-published rapid review vs. one journal-published systematic review.	Methods used by rapid review were abbreviated as compared with the systematic review. Even so, both reviews identified different trials with only 15% of trials included by both reviews; leading to conflicting conclusions by the two reviews.
**Warren 2007[32]**	Comparison of 39 interventional procedures evaluated by the British United Provident Association`s rapid appraisal algorithm vs. full National Institute for Health and Clinical Excellence health technology assessment.	Both rapid and full evaluations resulted in similar conclusions (90% of evaluations).

IQWiG = Institute for Quality and Efficiency in Health Care; NICE = National Institutes of Clinical Evidence; SMC = Scottish Medicines Consortium; STA = Single Technology Assessments; TAR = Technology Assessment Report

The conclusions of systematic reviews and rapid reviews on comparable topics were similar, with exceptions being attributed to the identification of different data sources (different included study designs and databases searched). In one study [31], both reviews identified different trials with only 15% overlap. This led to conflicting conclusions for the same research question. Furthermore, even though the conclusions markedly differed in only one comparison, there was overwhelming belief that systematic reviews provided a more trustworthy source of evidence because they included more outcomes, identified associations, confounding factors, and details in their final recommendations. Importantly, the assessment of the overall quality of the body of evidence (e.g. using GRADE or a similar tool) in rapid and systematic reviews was not compared in any study. It should be noted that we did not evaluate homogeneity in the research question(s), eligibility criteria or literature search period between rapid and systematic reviews.

KQ3: What are the potential biases associated with rapid reviews?

Following screening of 15,266 citations identified through the literature search, 33 methods studies were identified that investigated the potential biases that can be introduced into the review process “S3 Fig “. Two broad categories of evidence may address this question: evidence specific to rapid reviews, and evidence related to methodological approaches leading to bias whether employed in a rapid or systematic review. We were only able to identify evidence related to the latter.

One study [34] systematically searched for primary evidence on biases potentially introduced into the systematic review process. In addition, the overview by Tricco et al. [35] classified the reports as bias in identifying studies (sampling bias), choosing study biases, obtaining accurate data biases, and combining study bias. Gannon et al. [34] additionally found evidence on small and unpublished study biases. Methods studies highlighted potential biases that can be introduced from not including unpublished data [36,37], from industry sponsorship of individual studies [38], and from selective outcome reporting [39];Pandis, 2015; Tricco, 2016}.

Two publications [40,41] were updates of previously published methods studies [42,43]. One overview of reviews [35] identified ten published systematic reviews [40,42–49] examining biases related to searching for evidence (e.g. selective outcome reporting and publication bias). Furthermore, we identified additional recent systematic reviews [36,38,41] and methods studies [37,39,50,51] that confirm the presence of biases in the published literature. Recent studies investigating the level of publication bias occurring within systematic reviews are showing alarming results; with review authors, in many cases, not mentioning or evaluating the potential effect of publication bias on the results of their reviews [52,53]. An evaluation of such reviews by Onishi and Furukawa [54] highlighted the fact that readers should read the results of such reviews with caution. Even when review authors investigated sources of potential bias, it is often not taken into account when formulating the review’s conclusions [55].

Most systematic reviews are secondary research utilizing published and unpublished study reports, rather than individual patient data. Even so, it can be introduce bias when the study reports do not reflect all the study participants (e.g. excluding protocol violators or individuals that were lost for follow-up). In a recent studies, McCrae et al. [56] found that study eligibility criteria for primary study inclusion, search data restrictions and use of unpublished data was often not properly documented in published reviews. Further, Akl et al. [57] demonstrated that most systematic reviews do not check for, or account for, missing data. The authors also noted that this may lead to misleading judgements about the study-level risk of bias. Further, Page et al. [58,59] noted that selection criteria should not only be set for study selection, but also for effect estimates as they can bias the pooled effect estimates if chosen post-hoc.

An additional type of bias that is becoming more apparent is authorship bias. This can take many forms, but specifically with regards to systematic reviews, there is potential for confirmation bias when authors of reviews are themselves authors of the primary studies and/ or previous systematic reviews on the same topic [60] or have non-financial conflicts of interest [61].

## Discussion

Patients, clinicians, decision- and policy-makers at all levels of hospital administration, programme managers, regional authorities and officials at Ministries of Health are increasingly relying on systematic review evidence to support healthcare decisions. This reliance creates a challenge, since systematic reviews require a considerable investment in time and human resources.

There is consistent guidance available on how best to conduct systematic reviews [1,62,63]. Following this guidance often results in a review process lasting one year or longer [1,34]. Waiting for this length of time for information is not feasible in many decision-making contexts where decisions need to be made urgently, often in a matter of weeks, days, or even hours—hence the emergence of rapid reviews.

In contrast to systematic reviews, to date no widely agreed-upon methods to minimize the threats to the validity and credibility of rapid reviews have been established. Watt et al. [33] and Hailey et al. [64] reported varying definitions, purposes, and methods used by different groups conducting rapid reviews and rapid health technology assessments. Organizations surveyed reported a variety of tactics to expedite the review process, including restricting the scope of the research questions and using targeted and truncated search strategies. Even so, they concluded that the flexibility offered by rapid review methods allow meeting specific organizational needs for evidence-informed decision-making.

Harker et al. [65] noted that rapid reviews use a diverse set of methods that often differ from systematic reviews. This diversity makes evaluating recommendations from rapid reviews challenging as it is unclear what effect the potential biases introduced into the review process might have on the results [65,66]. Additionally, Gannon et al. [34] noted that because of the shortened timelines, rapid reviews may be prone to bias. As such, we recommend that any rapid review clearly describe the methods used, and the potential drawbacks of any deviations from accepted systematic review methods.

As rapid reviews are often considered to be “mini systematic reviews” or “rapidly conducted systematic reviews”, it would be of great interest to understand the trade-offs between comprehensiveness and timeliness, so that the importance of any potential bias can be gauged by the end user. Watt et al. [33] conducted a systematic search to identify studies comparing rapid reviews with more extensive reviews and was only able to identify two reports [67]. They observed, as we did, that most rapid and systematic reviews arrived at similar conclusions, even though different methods were used. The main differences between conclusions of a systematic and rapid review may be in the details of the methods used and extra information available in systematic reviews that is of greater interest to clinicians rather than to policy-makers [68]. We should emphasize that none of the reviewed comparative studies compared assessments of the strength of the bodies of evidence (or quality of evidence [69]) produced by systematic and rapid reviews. Nor did we formally appraise and compare the quality or validity of the systematic and rapid review evidence using tools like AMSTAR [3]. Also, it is not clear if this additional information provided by systematic reviews would have affected the decision-making process or result in any considerable fashion since decision-making requires additional information that is often not captured in rapid and systematic review conclusions.

For future research, it would be of value to not only investigate the conclusions from rapid and systematic review, but also the overall strength of the body of evidence that is collected for similar research questions. Since rapid review methods differ, it would be of benefit to determine how different short-cuts affect the overall strength of the evidence. This information may provide important insight for subsequent decision-making and further our understanding of the effects of procedures used to expedite the review process.

Systematic reviews are considered the best source of evidence to address uncertainties about healthcare alternative interventions [70]. Nevertheless, researchers recognized early that bias may enter the review process [71], potentially invalidating their findings. The most researched bias that can afflict the review process is publication bias. Our search for systematic reviews on biases that can be introduced into the review process identified a systematic review [34] and an overview of reviews [35]. Not surprisingly, the authors of the overview of reviews identified systematic reviews investigating publication bias only. Ganann et al. [34] also systematically searched for studies examining the implications of bias introduced into the review process through streamlined methods. Similar to our findings, they were unable to find any specific studies examining bias introduced by short-cutting the systematic review process, but they did identify kinds of potential bias that can be introduced into any systematic review due to publication bias (e.g. small and unpublished study biases and language of publication bias). That said, absence of evidence of bias in a rapid review process is not evidence of absence.

As for systematic reviews, concerns about the potential biases that can be introduced into the rapid review process are implied by theoretical considerations and have little empirical evidence to support them. For this reason, transparency in reporting rapid reviews methods is of the utmost importance to allow future methods research to confirm or refute claims regarding the impact of potential biases, and allow readers to assess for themselves the risk of bias in a given rapid review.

Assessing the validity of rapid reviews is often more difficult compared with systematic reviews as a diverse set of methodological approaches are adopted to conduct rapid reviews, some being more rigorous than others. Several biases may be encountered in less rigorous systematic reviews can also manifest in rapid reviews. For example, reviews that do not publish an a priori protocol may be more likely to have selective outcome reporting bias [72].

Since rapid reviews are commonly not just systematic reviews completed quickly (e.g. paucity of literature, or more reviewers involved), rapid reviews may benefit from having their own appraisal criteria above and beyond considerations for routine systematic reviews. For some rapid reviews, the bias may actually be less than that in a systematic review. For example, a rapid review that synthesizes evidence from both existing systematic reviews and recent primary literature may present a more trustworthy answer to the review question than would a previously published systematic review.

The strengths of this scoping review include the completeness of the search including searching multiple bibliographic databases and grey literature, using personal contacts and consultation with experts, and social media. We also used an a priori protocol and followed established methodological guidelines in the conduct and reporting of the systematic reviews conducted.

This report is not without limitations. With regards to the search strategies, we excluded non-English publications and restricted our literature search dates to increase the feasibility of this research. There is the potential that these decisions may have introduced biases into the search process. Further, though we attempted to collect information on current methods used by all groups conducting rapid reviews, our collection of information was incomplete. It is believed that details of most rapid reviews used for decision-making are not made publicly available. Also, since review methods may change over time, this information provides a snapshot only and may not reflect fully current or future practices.

In conclusion, the methods used to prepare rapid reviews are diverse, and context and organization specific. This report identified both similarities and differences between rapid and systematic review methods. Short-cuts could potentially fail to provide the usual rigor employed by systematic reviews. Albeit, existing comparisons of rapid and systematic reviews revealed little differences in their respective conclusions.

The results of this scoping review provided evidence on the state of rapid reviews to the WHO GRC. Additionally, they were used to support development of the new WHO guidance on producing evidence for Rapid Advice Guidelines [6]; including the recent rapid review on ‘Effectiveness of Personal Protective Equipment for Healthcare Workers Caring for Patients with Filovirus Disease’ [4].

## Supporting Information

S1 Fig Flow diagram for KQ 1—Methods used by organizations conducting rapid reviews. Modified PRISMA flow diagram showing the distribution of citations for KQ1.
(DOCX)

S2 Fig Flow diagram for KQ 2—Rapid reviews versus standard systematic reviews. Modified PRISMA flow diagram showing the distribution of citations for KQ2.
(DOCX)

S3 Fig Flow diagram for KQ 3 –Potential biases and confounding factors that can be introduced into a systematic review. Modified PRISMA flow diagram showing the distribution of citations for KQ3.
(DOCX)

S1 Table KQ1 search strategy Medline—Ovid format (7123 citations). Medline search strategy for KQ1.
(DOCX)

S2 Table KQ2 search strategy for Medline—Ovid format (2239 citations). Medline search strategy for KQ1.
(DOCX)

S3 Table KQ3 search strategy for Medline—Ovid format (6877 citations). Medline search strategy for KQ3.
(DOCX)

S1 Text Review protocol. Research protocol for this project.
(DOCX)

S2 Text Scoping review citations for all three scoping reviews. (ZIP)
