# Correction: Methods for Developing Evidence Reviews in Short Periods of Time: A Scoping Review

**DOI:** 10.1371/journal.pone.0172372

**Published:** 2017-02-13

**Authors:** Ahmed M. Abou-Setta, Maya M. Jeyaraman, Abdelhamid Attia, Hesham G. Al-Inany, Mauricio Ferri, Mohammed T. Ansari, Chantelle M. Garritty, Kenneth Bond, Susan L. Norris

The second author’s name is incorrect. The correct name is: Maya M. Jeyaraman. The correct citation is: Abou-Setta AM, Jeyaraman MM, Attia A, Al-Inany HG, Ferri M, Ansari MT, et al. (2016) Methods for Developing Evidence Reviews in Short Periods of Time: A Scoping Review. PLoS ONE 11(12): e0165903. doi:10.1371/journal.pone.0165903
