# Supplementary material for: Methods for Developing Evidence Reviews in Short Periods of Time: A Scoping Review
Source: PLoS One. 2016 Dec 8;11(12):e0165903. doi: 10.1371/journal.pone.0165903 (PMC5145149; doi:10.1371/journal.pone.0165903)
Supplement: S1 Fig — Modified PRISMA flow diagram showing the distribution of citations for KQ1. (DOCX) [file pone.0165903.s001.docx]

**S1 Fig. Flow diagram for KQ 1 - Methods used by organizations conducting rapid reviews**

Records excluded
(n = 10,007)

Full-text articles excluded: (n = 721)

Abstract only^*^ (n = 1)

Academic reviews (n = 613)

Not a rapid review (n = 103)

Non-English publication (n = 4)

Records identified through database searching

(n = 16,945 citations)

Additional records identified through other sources

(n = 768 citations)

Duplicate records removed
(n = 6,662)

Organizations conducting

rapid reviews

(n = 65)

Methods used by organizations conducting rapid reviews

(n = 29)

Records identified through all sources
(n = 17,713)

Unique records screened
(n = 11,051)

Full-text articles assessed for eligibility
(n = 1,044)

Organizational rapid reviews

(n = 323)

Organizations excluded: (n = 45)

Declined participation in survey (n = 3)

Did not respond to survey & rapid review methods not found online (n = 42)

^*^ Not enough information to make a decision on inclusion.
