# Supplementary material for: Methods for Developing Evidence Reviews in Short Periods of Time: A Scoping Review
Source: PLoS One. 2016 Dec 8;11(12):e0165903. doi: 10.1371/journal.pone.0165903 (PMC5145149; doi:10.1371/journal.pone.0165903)
Supplement: S2 Fig — Modified PRISMA flow diagram showing the distribution of citations for KQ2. (DOCX) [file pone.0165903.s002.docx]

**S2 Fig. Flow diagram for KQ 2 - Rapid reviews versus standard systematic reviews**

Full-text articles excluded:

Non-English publication (n = 1)

No comparison of rapid vs. full SR (n = 22)

Unavailable via library services (n = 9)

Records excluded
(n = 5,852)

Records identified through database searching

(n = 8,796 citations)

Additional records identified through other sources

(n = 20 citations)

Duplicate records removed
(n = 2,922)

Records identified through all sources
(n = 8,816)

Unique records screened
(n = 5,894)

Full-text articles assessed for eligibility
(n = 42)

Rapid vs. full SR comparisons (n = 10)

Primary publications (n = 9)

Companion publications (n = 1)
