# Supplementary material for: Methods for Developing Evidence Reviews in Short Periods of Time: A Scoping Review
Source: PLoS One. 2016 Dec 8;11(12):e0165903. doi: 10.1371/journal.pone.0165903 (PMC5145149; doi:10.1371/journal.pone.0165903)
Supplement: S3 Fig — Modified PRISMA flow diagram showing the distribution of citations for KQ3. (DOCX) [file pone.0165903.s003.docx]

**S3 Fig. Flow diagram for KQ 3 – Potential biases and confounding factors that can be introduced into a systematic review**

Records excluded
(n = 15,103

Full-text articles excluded:

Does not meet inclusion criteria (n = 130)

Records identified through database searching

(n = 16,204 citations)

Additional records identified through other sources

(n = 10 citations)

Duplicate records removed
(n = 948)

Records identified through all sources
(n = 16,214)

Unique records screened
(n = 15,266)

Full-text articles assessed for eligibility
(n = 163)

Included studies (n = 33)

Systematic reviews (n = 15)

Recent methods studies (n = 18)
