# Supplementary material for: Methods for Developing Evidence Reviews in Short Periods of Time: A Scoping Review
Source: PLoS One. 2016 Dec 8;11(12):e0165903. doi: 10.1371/journal.pone.0165903 (PMC5145149; doi:10.1371/journal.pone.0165903)
Supplement: S2 Table — Medline search strategy for KQ1. (DOCX) [file pone.0165903.s005.docx]

**S2 Table. KQ2 search strategy for *Medline* – Ovid format (2239 citations)**

1. accelerated review$.ti,ab.
2. archimedes.ti,ab.
3. (best bet or best bets).ti,ab.
4. best evidence topic$.ti,ab.
5. expedited review$.ti,ab.
6. hospital-based review$.ti,ab.
7. hospital-based systematic review$.ti,ab.
8. meta-evaluation$.ti,ab.
9. meta-method$.ti,ab.
10. mini-HTA.ti,ab.
11. mini health technology assessment$.ti,ab.
12. (rapid and (guideline$ or realist or review$ or systematic$)).ti,ab.
13. rapid advice.ti,ab.
14. rapid assessment$.ti,ab.
15. rapid evidence.ti,ab.
16. rapid review$.ti,ab.
17. rapid search$.ti,ab.
18. rapid synthesis.ti,ab.
19. rapid technology assessment$.ti,ab.
20. rapid update$.ti,ab.
21. short-cut review$.ti,ab.
22. or/1-21
23. exp clinical practice guideline/
24. exp guideline/
25. exp guidelines as topic/
26. exp review literature as topic/
27. exp technology assessment, biomedical/
28. systematic$.ti,ab.
29. or/23-28
30. 22 and 29
31. limit 30 to yr="1980 -Current"
32. limit 31 to English language
33. (versus or vs).ti,ab.
34. compar$.ti,ab.
35. contrast.ti,ab.
36. or/33-35
37. 32 and 36
