# Supplementary material for: Methods for Developing Evidence Reviews in Short Periods of Time: A Scoping Review
Source: PLoS One. 2016 Dec 8;11(12):e0165903. doi: 10.1371/journal.pone.0165903 (PMC5145149; doi:10.1371/journal.pone.0165903)
Supplement: S3 Table — Medline search strategy for KQ3. (DOCX) [file pone.0165903.s006.docx]

**S3 Table. KQ3 search strategy for *Medline* – Ovid format (6877 citations)**

1. exp "bias (epidemiology)"/
2. publication bias/
3. selection bias/
4. bias$.ti,ab.
5. ecological fallac$.ti,ab.
6. outcome measurement error$.ti,ab.
7. sampling error$.ti,ab.
8. or/1-7
9. exp clinical trial/
10. exp clinical trials as topic/
11. or/9-10
12. 8 not 11
13. exp meta-analysis/
14. meta-analys$.ti,ab.
15. systematic$ review$.ti,ab.
16. or/13-15
17. 12 and 16
18. exp animals/ not humans.sh.
19. 17 not 18
20. limit 19 to English language
21. limit 20 to yr="1980 -Current"
