# Supplementary material for: Methods for Developing Evidence Reviews in Short Periods of Time: A Scoping Review
Source: PLoS One. 2016 Dec 8;11(12):e0165903. doi: 10.1371/journal.pone.0165903 (PMC5145149; doi:10.1371/journal.pone.0165903)
Supplement: S1 Text — Research protocol for this project. (DOCX) [file pone.0165903.s007.docx]

**S1Text. Review protocol.**

**Objective:**

The objective of this review will be to systematically search for, identify, and summarize the: (a) methods for producing systematic reviews and guidelines rapidly currently used by organizations around the world, (b) evidence on the reliability, validity, and quality of these ‘rapid’ reviews/ guidelines compared with ‘full’ reviews/ guidelines, and (c) sources of potential bias associated with methods used in order to complete a “rapid” review/ guideline development. Even though the exact definition of “rapid” in this context varies from organization to organization, we are interested in evidence reviews and guidelines that are conducted within three months or less by limiting scope or methods traditionally used in “full” systematic reviews (e.g. searching only one database, having one reviewer screen/ extract data, etc.) or deviations from an organization’s standards for “full” review preparation. The ultimate goal will be to inform the WHO GRC about the processes and procedures that might be considered reasonable to modify when they are required to develop evidence summaries and subsequent guidance within three months or less.

**Methods:**

As this review spans several separate but closely related key questions (KQ), we will conduct separate reviews for each key question; each tailored to maximize the effectiveness of the review process. The search strategies were developed by a team of experienced researchers and search coordinators and were peer-reviewed (Relevo, AHRQ, 2012). The protocols for each KQ will be published publicly in accordance with the current academic standards of literature reviewing and reporting (Moher, J Clin Epidemiol, 2009; Institutes of Medicine, 2011).

***Search strategy for identification of publications***

*KQ1: Research Methods for Producing Systematic Reviews and Guidelines Rapidly*

We will conduct a systematic scoping search in Cochrane Library (Cochrane Reviews and non-Cochrane Reviews) (Wiley), Embase (Ovid), and Medline (Ovid) (Appendix 2) for systematic reviews and guidelines produced rapidly (Appendix 1). The searches will be limited to English language articles published since the year 1980 for feasibility, and since it is not anticipated that searching prior to this date or in additional languages will bias the search results. These assumptions are built on the fact that most citations and core journals in Medline are published in the English language, most organizations commonly known to regularly conduct systematic reviews and guidelines publish some version of their reports in the English language, and even though there may be systematic reviews and guidelines published several decades ago, the accepted standards for the conduct of systematic reviews and practice guidelines have changed markedly over the years; making the importance of legacy methods of little importance to the current project.

*KQ2: Reliability, Validity, and Quality of Systematic Reviews and Guidelines Produced Rapidly*

To identify studies that assessed the reliability, validity or quality of rapidly-produced versus standard “full” reviews or guidelines, we will use a similar search strategy as presented for KQ1, but limit the search to comparative studies (Appendix 2). We will also search the Grey Matters’ search list (Canadian Agency for Drugs and Technologies in Health, 2013). The same language and date limits described for KQ1 will be applied.

*KQ3: Potential Biases Associated with Producing Systematic Reviews and Guidelines Rapidly*

To identify studies that investigated and/or provided empirical evidence of the effect of potential biases on the results of systematic reviews and guidelines, we will use a series of searches in the Cochrane Library (including the Cochrane Methodology Register) (Wiley), Embase (Ovid), and Medline (Ovid) (Appendix 3) for the following classifications of potential sources of biases:

- scope,
- sources of evidence,
- searching strategies,
- data abstraction,
- data analysis,
- assessment of quality of individual studies,
- assessment of the strength or quality of a body of evidence.

Biases are anticipated to be introduced by deviations from standard ‘full’ standard systematic reviews and guideline preparation protocols, and all identified biases will be evaluated. Since a lot of secondary research has already been conducted and published on this topic, we will begin with a search for systematic reviews since 1980, with the addition of primary literature from 2010 onwards. The limitations on searching the literature to answer this question are both for feasibility and, as with the search strategies for KQ1 and KQ2, we do not anticipate that searching for legacy publications would be of added value for the current review. The search for additional recent publications will allow the capture of additional reports that might not have been captured in previously completed reviews.

For all key questions, in addition to the electronic bibliographic searches, we will attempt to identify unpublished and non-indexed documents through personal contact and consultation with experts soliciting citations and documents on social media (e.g. LinkedIn Evidence-Based Medicine groups) and an Email listserv (evidence-basedhealth@ jiscmail.ac.uk) and using a snowballing sampling technique. The reference lists of relevant articles will also be searched for relevant citations; a process commonly referred to as “pearling”. Reference management for all searches (KQ1-3) will be done using EndNote™ (version X5, Thomson Reuters, Carlsbad, CA, USA).

***Study selection***

*KQ1: Research Methods for Producing Systematic Reviews and Guidelines Rapidly*

The inclusion criteria are systematic reviews or guidelines produced rapidly (3 months or less), conducted by an organization to inform decision making (i.e. not only for academic purposes), and using “current” methods. By “current” methods we mean those reflected in the work being published within the last five years or methods currently used by the organization that conducted the systematic review or guideline (confirmed by, e.g., online documentation or confirmed via personal contact). In the event we find older articles of interest, we will attempt to contact the corresponding author or organizational representative to determine if these methods are still being used today.

*KQ2: Reliability, Validity, and Quality of Systematic Reviews and Guidelines Produced Rapidly*

The inclusion criteria are studies comparing systematic reviews or guidelines produced rapidly (3 months or less) with standard ‘full’ systematic reviews and guidelines, and reporting on the comparative reliability, validity and/ or quality of the evidence summary. These studies may have used abbreviated methods (e.g. limiting the number of databases searched) or used accelerated methods (e.g. increasing the number of reviewers on the team in order to complete the tasks faster).

*KQ3: Potential Biases Associated with Producing Systematic Reviews and Guidelines Rapidly*

The inclusion criteria are systematic reviews and primary studies investigating potential biases that may be introduced into systematic reviews or guidelines as a result of deviating from standard “full” systematic review and guideline methods. Potential sources of biases to consider include, but are not restricted to limits in:

- scope,
- sources of evidence,
- searching strategies,
- data abstraction,
- data analysis,
- assessment of quality of individual studies,
- assessment of the strength or
- quality of a body of evidence.

For KQ1, in accordance to scoping review methodology, study screening and selection will be conducted by one reviewer using standardized and piloted screening forms. For KQ2 and KQ3, we will use a two-stage process for study screening and selection using standardized and piloted screening forms. Two reviewers will independently screen the titles and abstracts of search results to determine if a citation meets the inclusion criteria. The full text of citations classified as “include” or “unclear” will be further reviewed with reference to the predetermined inclusion and exclusion criteria. Discrepancies between the two reviewers will be resolved through consensus by discussion with a third reviewer, as required.

***Data abstraction and management***

All data from included study reports will be abstracted using standardized and pre-tested data extraction forms. For KQ1, methods used by organizations for conducting rapid reviews/ guidelines will be extracted by one reviewer, or provided directly by an organizational representative (e.g. personal contact). For KQ2 and KQ3, one reviewer will extract data, using a standardized and pre-tested data extraction forms, from included reports. A second reviewer will review the extractions for completeness and accuracy. Discrepancies between the two reviewers will be resolved through consensus and discussion with a third reviewer, as required. In the case, data is provided via personal contact, one reviewer will review the information for completeness. Data management will be performed using Microsoft Excel™ 2010 (Excel version 14, Microsoft Corp., Redmond, WA, USA).

**Analyses:**

*KQ1: Research Methods for Producing Systematic Reviews and Guidelines Rapidly*

Data gathered on the methods used by organizations conducting rapid reviews and guidelines will be classified and categorized in order to generate a map of the methods used worldwide. We will provide descriptive statistics on the frequency and density distributions of the used methods in order to determine prevalence and trends in the data.

*KQ2: Reliability, Validity, and Quality of Systematic Reviews and Guidelines Produced Rapidly*

We will summarize the objectives, methods used, and results of studies comparing rapid with full reviews and guidelines. No meta-analytic techniques will be used.

*KQ3: Potential Biases Associated with Producing Systematic Reviews and Guidelines Rapidly*

We will classify and categorize identified potential biases and summarize their estimated effects on the ‘true’ effect estimate. Meta-analysis and/or meta-regression will be conducted if adequate data is available.
